# Atypical Topographical Organization of Global Form and Motion Processing in 5-Month-Old Infants at Risk for Autism

**DOI:** 10.1007/s10803-020-04523-2

**Published:** 2020-05-26

**Authors:** Pär Nyström, Emily Jones, Fahimeh Darki, Sven Bölte, Terje Falck-Ytter

**Affiliations:** 1 Uppsala Child & Babylab, Department of Psychology, Uppsala University, Box 1225, 75142 Uppsala, Sweden; 2 Centre for Brain & Cognitive Development, Birkbeck, University of London, London, UK; 3 Karolinska Institutet Center of Neurodevelopmental Disorders (KIND), Centre for Psychiatry Research, Department of Women’s and Children’s Health, Karolinska Institutet & Stockholm Health Care Services, Region Stockholm, Stockholm, Sweden; 4 Child and Adolescent Psychiatry, Stockholm Health Services, Region Stockholm, Stockholm, Sweden; 5 Curtin Autism Research Group, School of Occupational Therapy, Social Work and Speech Pathology, Curtin University, Perth, WA Australia; 6 Swedish Collegium for Advanced Study (SCAS), Uppsala, Sweden

**Keywords:** Autism, Infants, EEG, Global coherence, Motion, Developmental disorders

## Abstract

Research indicates that individuals with autism spectrum disorder (ASD) are superior at local processing while the integration of local features to global percepts is reduced. Here, we compared infants at familiar risk for ASD to typically developing infants in terms of global coherence processing at 5 months of age, using steady state visually evoked potentials (SSVEP). We found a different topographical organization for global form and motion processing in infants at risk (n = 50) than in controls (n = 23). In contrast, activation patterns for local visual change were strikingly similar between groups. Although preliminary, the results represent the first neurophysiological evidence supporting the view that basic atypicalities in perception may play a role in the developmental pathways leading to ASD.

**Electronic supplementary material:**

The online version of this article (10.1007/s10803-020-04523-2) contains supplementary material, which is available to authorized users.

## Introduction

Autism spectrum disorder (ASD) is a highly heritable neurodevelopmental condition affecting up to 1–1.5% of the population. In addition to the characteristic impairments in social communication and inflexible behavior patterns, early atypicalities in perception and sensory processing are increasingly acknowledged as crucial for our understanding of the neurobiology of the condition (Robertson and Baron-Cohen 2017; Falck-Ytter et al. 2018; Nyström et al. 2018). One relatively consistent finding in previous research is that individuals with ASD are superior at local processing, while the integration of coherent local features to a global percept is reduced (Bertone et al. 2003), suggesting alterations in the magnocellular pathway (Milne et al. 2002; McCleery et al. 2007) and the dorsal stream (Pellicano et al. 2005; Spencer et al. 2000). This may in turn impact a broad range of developmental processes, such as face processing, action perception, motor development (sitting crawling, eye/hand coordination), which all may contribute to the ASD phenotype. In typical development, global form and motion coherence processing develops during the first 6 months of life (Wattam-Bell et al. 2010; Braddick et al. 2003). It is possible that atypicalities in these fundamental perceptual functions are present before the emergence of other symptoms—a finding that would have important implications for developmental theories of ASD, and in the current debate between social-first and domain general theories. In this study, we used electroencephalogram (EEG) and a steady state visually evoked potential (SSVEP) paradigm to map the activity in primary and extrastriate areas that integrate outputs from the primary visual cortex (Wattam-Bell et al. 2010) and determine whether processing of global form or motion coherence are impaired in infants at risk for ASD. We assessed infants with an older full sibling with ASD because of the high heritability: these infants are at high risk for developing ASD and related neurodevelopmental problems themselves (roughly 20% develop ASD, but as many as 50% develop symptoms that motivate clinical evaluation (Ozonoff et al. 2014)).

## Method

Participating families were recruited within the Early Autism Sweden (EASE) project, a longitudinal study of infants at risk for autism (using a prospective sibling design, Nyström et al. 2018, 2019; Falck-Ytter et al. 2018). Participants were infants with one or more siblings with an ASD diagnosis to a high risk group (HR, n = 63, final sample n = 50) and comparable low risk control infants (LR, n = 28, final sample n = 23) that had at least one typically developing older full sibling and no first or second degree relatives with ASD. Families with infants in the HR group had been contacted through advertisements, the project’s web site and from clinical units. The older sibling diagnosis was confirmed by inspection of clinical records. Infants in the LR group were recruited from population birth records in selected municipalities in the larger Stockholm area (about 20% respond to our recruitment letters), and had at least one typically developing older full sibling and no first or second degree relatives with ASD. Infants with visual or auditory impairments or with known medical conditions (including prematurity before week 36) or genetic syndromes were excluded. The EEG and the Mullen Scales of Early Learning (MSEL) was recorded at 5 months of age. The HR and LR groups were matched according to gender, age, MSEL at 5 months and socioeconomic background (see Table 1).

**Table 1:** Background characteristics of the groups

	LR group	HR group	p-values
Nr subjects	23 (14 girls)	50 (24 girls)	χ^2^ = 1.045, p = > .25
Age in days	163.435 (14.254)	164.080 (17.438)	t(71) = − 0.155, p > .25
MSEL^a^	100.652 (7.935)	97.000 (11.181)	t(71) = 1.409, p = 0.163
MSEL_NVIQ^b^	111.522 (18.075)	107.940 (24.348)	(71) = 0.629, p > .25
MSEL_VIQ^c^	100.522 (19.409)	95.980 (18.776)	t(71) = .950, p > .25
MSEL_GM^d^	49.000 (7.663)	48.020 (7.821)	t(71) = .500, p > .25
MSEL_VR^e^	53.130 (8.449)	52.480 (10.238)	t(71) = .266, p > .25
MSEL_FM^f^	48.609 (7.680)	45.960 (9.238)	t(71) = 1.197, p = 0.235
MSEL_RL^g^	51.565 (8.117)	48.220 (7.797)	t(71) = 1.681, p = 0.097
MSEL_EL^h^	47.826 (6.365)	47.360 (6.369)	t(71) = .291, p > .25
SES^i^	0.052 (0.756)	– 0.126 (0.853)	t(61) = .797, p > .25

^a^MSEL composite score

^b^Nonverbal IQ subscale

^c^Verbal IQ subscale

^d^Gross motor subscale

^e^Visual reception subscale

^f^Fine motor subscale

^g^Receptive language subscale

^h^Expressive language subscale

^i^Socioeconomic status, calculated on the basis of parental education and income (equal weight), expressed as *z*-score (for this measure nine families did not disclose this information)

Written informed consent was collected from all parents. The study was approved by the Ethics Board in Stockholm and conducted in accordance with the 1964 Declaration of Helsinki.

### Procedure

Families were welcomed upon arrival and given verbal instructions of the tasks during the day. Different assessments were performed at the different time points; see Nyström et al. (2017, 2018, 2019) and Falck-Ytter et al. (2018) for other experimental tasks during the day. The MSEL assessment was always performed by an experienced clinician before lunch.

The EEG was recorded using an age appropriate 128-channel Geodesic Sensor Net (Age appropriate 128-channel Geodesic Sensor Nets (HCGSN 130; EGI, Eugene, OR). The signal was sampled at 500 Hz relative to the vertex reference, amplified by EGI Net amplifier (GES 300 Amp; EGI, Eugene, OR) and stored for off-line analysis.

**Fig. 1:**
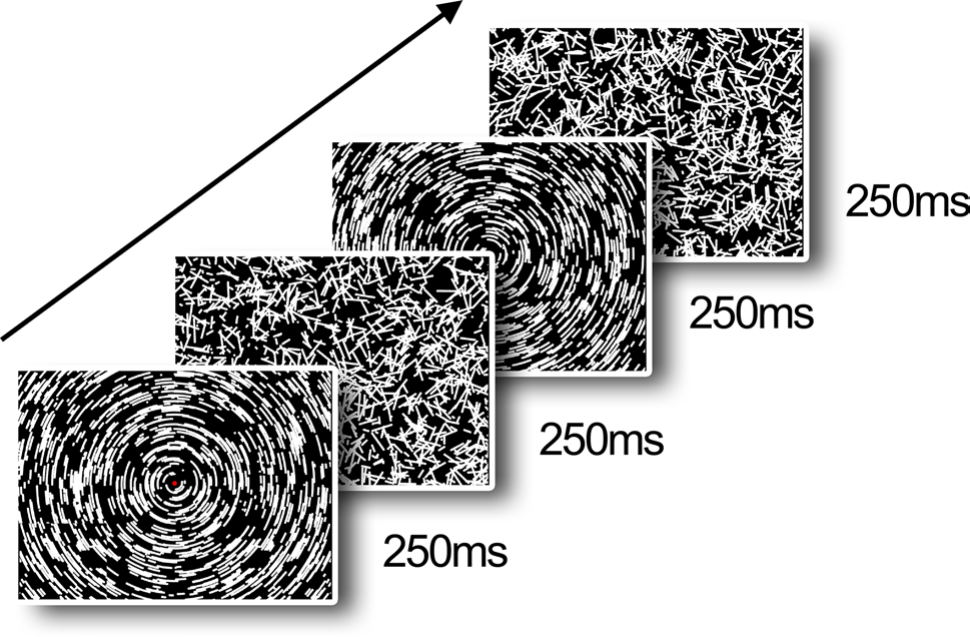
Stimuli from our VERP (visual event related potential) experiment

Stimuli were generated by a MacBook Pro using the PsychToolbox in MATLAB (2013a), running under OS X EL Capitan (version 10.11.6), and presented on a BenQ (23.5 inches) monitor with 1920 × 1080 pixel resolution operating at 60 Hz frame rate. As in a previous study (Wattam-Bell et al. 2010), for both form and motion, 2000 local arcs were always present on screen, alternating between coherent motion/form and random coherent displacement every 250 ms. Each local arc consisted of eight white dots plotted on a dark background (0.29° visual degrees). Following an 8-frame lifetime, each dot was replotted in a fresh random location on the screen. When plotted simultaneously on the screen, these dots created a short, static arc segment (the form condition). When plotted successively, they create a brief sample of motion along an arc trajectory for the motion condition (displacement between frames gave a speed of 8.6 visual degrees/sec). In the form condition the coherent interval resulted in a global concentric texture (see Fig. 1 for an example), and in the motion condition the coherent interval created a globally rotating motion about a common origin at the center of the screen. Patterns were viewed at ~ 60 cm and subtended 47.4° × 27.8°. The stimuli were presented in blocks with a duration of 12 s, containing 24 cycles. Each cycle had both a random phase (250 ms) and coherent phase (250 ms); see supplementary materials for video examples. Each 12 s block contained only form or motion stimuli, to entrain brain responses to the frequency of the specific condition, and the blocks were interleaved with unrelated experimental stimuli. We presented 10 form blocks and 10 motion blocks, giving 240 cycles in total for each condition.

### Analysis

All analysis was done using MATLAB (R2018b), the EEGLAB toolbox (Delorme and Makeig 2004), and the TimeStudio scientific workflow system (Nyström et al. 2015). A subset of 121 EEG channels covering most of the scalp were used for analysis. All channels were resampled to 100 Hz to reduce computer memory load, and were high pass filtered at 0.5 Hz to filter out slow drifts in the signal. All channels were then re-referenced to average reference, and segmented into stimuli cycles as described above. To exclude artifacts, all cycles with a voltage range exceeding 100uV were excluded, as well as the first and last second of the block.

To extract brain responses related to the stimuli we calculated the T2circ statistics for all cycles and all channels separately. The T2circ statistics is based on both the real and imaginary coefficients of a Fourier transform for the frequency of the stimuli (or any other specified frequency), and requires systemic responses in both the amplitude and phase domain (Victor and Mast 1991). A statistically significant signal at the fundamental stimulus frequency (2 Hz) was taken as evidence for a neural process sensitive to global coherence, and because there are twice as many global changes every cycle (from random to coherent, and from coherent to random), a statistically significant signal at the double frequency (4 Hz) was taken as evidence for neural processes sensitive to low level contrast changes, as in Wattam-Bell et al. (2010).

All subjects without any significant channel, as tested with the T2circ statistics using all cycles in each channel, were excluded from further analysis. After exclusion, in the global motion condition the HR group (n = 50) contributed a mean of 155.2 (SD = 45.9) cycles, and the LR group (n = 23) a mean of 164.0 (SD = 38.5) cycles. In the global form condition the HR group (n = 50) contributed a mean of 155.6 (SD = 44.0) cycles, and the LR group (n = 23) mean = 162.7 (SD = 37.9) cycles.

**Fig. 2:**
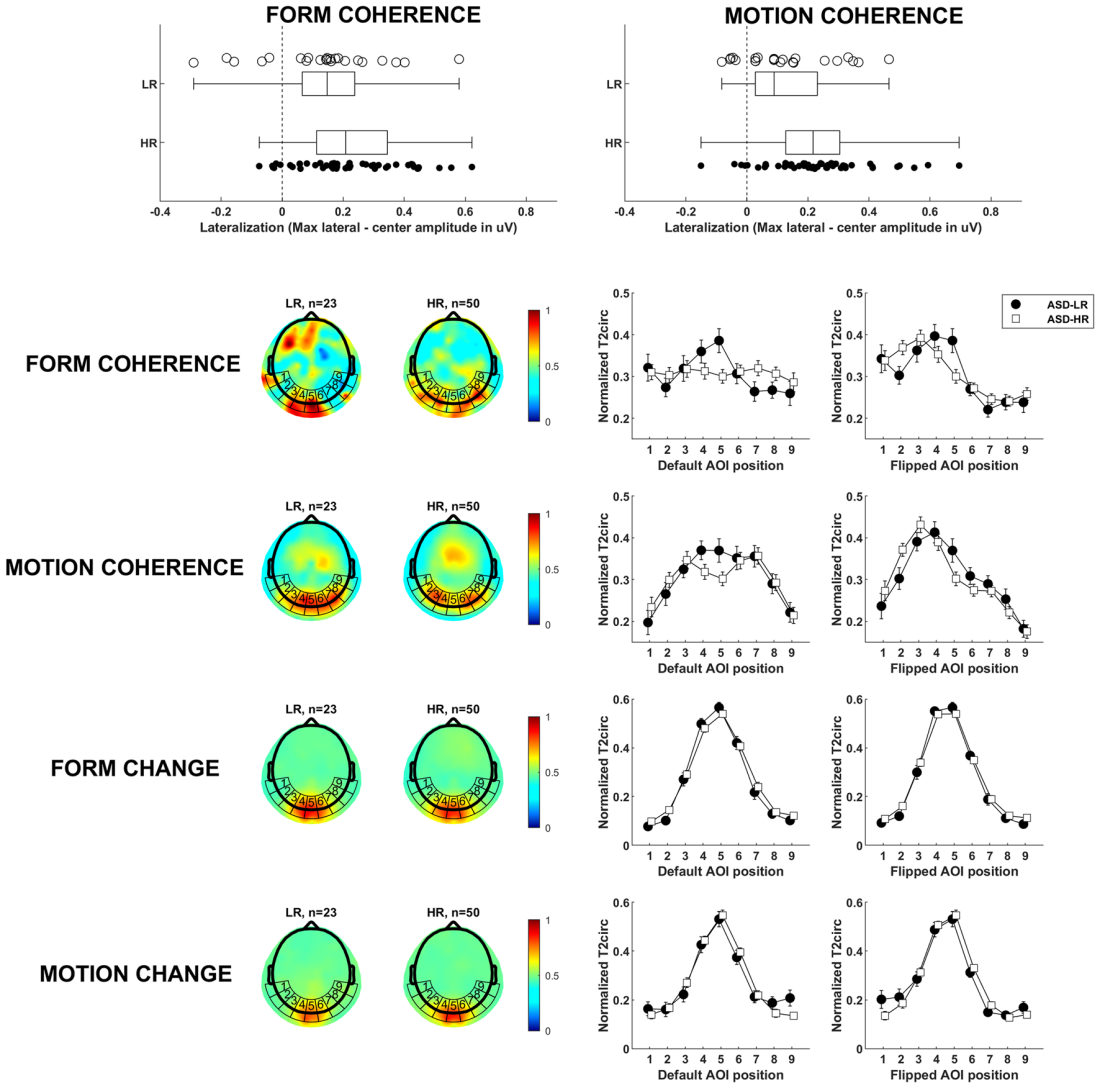
Main results. Row 1: Compared to typical controls, infants at risk for ASD have more lateralized brain activation when processing globally coherent visual information. Markers denote individual infants. The edges of the box are the 25th and 75th percentiles, and the whiskers extend to the most extreme data points. Row 2 and 3: To the left, the mean topographical plots for the LR and HR groups, with AOI positions superimposed. The heatmap show T2circ-statistics as a measure of brain activation. To the right, the activation profiles for the AOIs’ average values. The plot with default AOI positions show the collected data, untransformed. The plot with flipped AOI positions show data where individuals with activation maxima in the right hemisphere have been horizontally flipped (i.e. use positions 9–1 instead of 1–9), to highlight the general lateralization pattern across individuals. Row 4 and 5: When processing lower level changes of the stimuli, both groups show similar centralized topographical plots and activation profiles, presumably V1

Statistical comparison of topographical distributions was based on T2circ values by interpolating the electrode values over a uniform grid (~ 3500 vertices), bounded by the outer electrodes of the nets. These interpolated values were averaged separately within nine areas of interest (AOIs) around the back of the head (see Fig. 2, row 2–5). In the previous study by Wattam-Bell et al. (2010) only five AOIs were used for the same scalp surface, but we increased the number of AOIs to get a more detailed spatial profile, and less risk of pooling different brain processes into the same AOI. At this stage, each subject’s data consisted of separate 9-point spatial brain activity profiles. The activity profiles were vector-normalized (McCarthy and Wood 1985) to eliminate overall amplitude differences between individual infants. Our dependent measure used in our main analysis was a measure of the topographic centralization of the signal, calculated by subtracting the central AOI activation from the maximum activation in all AOIs. To visualize the general central vs lateral topographic pattern across individuals, the default AOI positions were flipped along the sagittal plane so that the maximum lateral amplitude always was presented on the left hemisphere (Nb participants flipped: LR form n = 9/23, HR form n = 26/50, LR motion n = 15/23, HR motion n = 30/50). The number of hemispherical flips did not differ between groups (χ^2^ test, group*condition, p > .25). This way left and right laterality between infants cannot cancel out in the average response. The resulting activity profiles for global change/coherence and form/motion profiles are shown in Fig. 2.

## Results

Local visual processing of simple form/motion change (4 Hz activity), which is very likely to reflect activity in V1 (Wattam-Bell et al. 2010), did not differ between groups: a repeated measures ANOVA testing for differences in activity level in the center AOI, with condition (form/motion) as within-subject variable and group as between subject variable, showed no significant main effects of group, F(1, 71) = .035, p > .25, or condition, F(1, 71) = .623, p > .25, and there was no interaction effect, F(1, 71) = 1.296, p > .25. The groups’ similar activity profiles (Fig. 2, row 4–5), also suggests that the first levels of visual processing is intact in the HR group: a repeated measures ANOVA testing the topographic centralization showed no significant main effect of group, F(1, 71) = .034, p > .25, or condition, F(1, 71) = .019, p > .25, and there was no significant interaction effect, F(1, 71) = 1.323, p > .25.

In contrast, at the coherence processing level (2 Hz activity), profiles diverged between groups (Fig. 2, row 2–3). A repeated measures ANOVA which included condition (form/motion) as within-subject variable and group as between subject variable showed a significant main effect of group, with more lateral activation in the HR group [F(1, 71) = 11.13, p = .001, η^2^ = .135; HR n = 50, form M = .229, motion M = .229; LR n = 23, form M = .136, motion M = .125, see Fig. 2, row 1]. No interaction effect (p > .25) or main effect of condition (p > .25) was found, supporting the notion that the results reflect alterations in integrative processes of coherent visual information in general (both form and motion).

Results for separate ANOVAs for the form and motion conditions show differences between the HR and the LR groups to motion coherence [F(1, 71) = 6.639, p = .012, η^2^ = .086], with more lateral patterns of activation in the HR group (see Fig. 2, row 2). For form, the pattern was similar, with a significant difference between groups [F(1, 71) = 4.360, p = .040, η^2^ = .058].

To investigate differences in topological responses in the global coherence conditions (2 Hz) between groups in more detail, an exploratory ANOVA tested the distance between the center AOI and the lateral AOI with the maximum T2circ value, using the AOI position index as the Euclidian position. There was a main effect of group, with more lateral peak responses in the HR group [F(1, 71) = 4.282, p = .042, η^2^ = .057; HR n = 50, form M = 2.240, motion M = 2.020; LR n = 23, form M = 1.870, motion M = 1.522]. There was no main effect of condition (form/motion), p = .243, and no interaction effects between group and condition, p > .25. When splitting this ANOVA by condition, the motion condition was marginally significant group difference, [F(1, 71) = 3.202, p = .078, η^2^ = .043], but the form condition was not significant, p > .25.

## Discussion

A large body of research show that individuals diagnosed with ASD have a detail-focused processing style, possibly at the expense of global information processing (Bolte et al. 2007; Bertone et al. 2003; Pellicano et al. 2005). This study is the first to suggest that similar atypicalities may be present in infants at risk for ASD already at 5 months—i.e. long before behavioral symptoms of ASD are observable. That the two conditions (form and motion) gave rise to very similar group differences indicates that both dorsal and ventral streams (Milne et al. 2002; Bertone et al. 2003; Wattam-Bell et al. 2010; Pellicano et al. 2005) are involved.

Importantly, the results for local motion/form change (Fig. 2) was strikingly similar, which suggests that group differences were related to global coherence processing. Effective perception of global configurations is critical for further development [e.g. the development of effective face processing (McCleery et al. 2009)], and the current findings can generate important new leads for future longitudinal studies.

Because the EEG methodology only measure the scalp, it is difficult to exactly pinpoint the processing sources in terms of undisputable brain areas. Yet, the topological activation patterns and the significant group differences in maximum response position suggest that the HR group activate lateralized brain areas such as V3 and V5/MT (Wattam-Bell et al. 2010) more than the LR group when observing coherent visual form and motion. These alterations may be functionally important and have behavioral effects throughout development, and they are also informative of neural processing and possibly also neural connectivity in the early etiology of ASD. However, due to constraints of the EEG method and the lack of diagnostic outcome, the current findings should be complemented with longitudinal neural and behavioral measures before the impact of the current findings can be evaluated.

In comparison to previous work suggesting a “dorsal-stream vulnerability” (Atkinson and Braddick 2011; Braddick et al. 2003; Spencer et al. 2000), and early alterations in the magnocellular pathways (McCleery et al. 2007) in ASD, our results show differences in both dorsal and ventral processing. However, we have only studied one time point, and it is possible that longitudinal studies beyond infancy would find different developmental pathways for the dorsal and ventral stream. For the time being, we consider our results compatible with, but not necessarily supportive of, a general dorsal-stream vulnerability, and we suggest that ventral-stream alterations may be equally relevant to study.

An important finding in our study is the distinct shift from intact neural processing at low level (change) to altered lateralization for higher level processing (coherence) in the HR group. Future studies could therefore, based on the current findings, increase spatial resolution in indicated regions and possibly also add information from magnetic resonance imaging (MRI), to better locate where and how different neurodevelopmental groups diverge.

A significant limitation of the current study is that we do not yet have diagnostic outcome data from the samples. This entails that it is difficult to say whether the atypical patterns are specifically related to ASD or not. It is not uncommon that antecedent markers of ASD are both linked to diagnosis categorically, but also to symptom strength dimensionally in the whole HR (and sometimes LR) sample (Nyström et al. 2018, 2019). Future studies should thus link neurophysiological profiles such as those presented here to later developmental outcomes. Next, our study only assesses visual processing with EEG at 5 months, but longitudinal assessment of visual processing would be needed to clarify whether the observed alteration in HR is an altered mechanism or an immature response, and when differences are present.

In conclusion, this study showed that there are neurophysiological alterations in very basic perceptual processes in infants at high risk for ASD already at 5 months of age. If corroborated by further studies linking this finding directly to ASD diagnosis, it may have far-reaching consequences for our understanding of developmental mechanisms in infants with the condition.

## Electronic supplementary material

Below is the link to the electronic supplementary material.

Supplementary file1 (MOV 14031 kb)

Supplementary file2 (MOV 20690 kb)
